# Molecular Tools for the Selective Detection of Nine Diatom Species Biomarkers of Various Water Quality Levels

**DOI:** 10.3390/ijerph120505485

**Published:** 2015-05-22

**Authors:** Lucia Cimarelli, Kumar Saurabh Singh, Nguyen Thi Nhu Mai, Bidhan Chandra Dhar, Anna Brandi, Letizia Brandi, Roberto Spurio

**Affiliations:** Laboratory of Genetics, School of Biosciences and Veterinary Medicine, University of Camerino, 62032 Camerino, Italy; E-Mails: lucia.cimarelli@studenti.unicam.it (L.C.); kumar.saurabhsingh@unicam.it (K.S.S.); nhumai7475@yahoo.com (N.T.N.M.); bidhan.dhar@gmail.com (B.C.D.); anna.brandi@unicam.it (A.B.); letizia.brandi@unicam.it (L.B.)

**Keywords:** diatoms, freshwater, ecological status, oligonucleotide probes, microarrays

## Abstract

Our understanding of the composition of diatom communities and their response to environmental changes is currently limited by laborious taxonomic identification procedures. Advances in molecular technologies are expected to contribute more efficient, robust and sensitive tools for the detection of these ecologically relevant microorganisms. There is a need to explore and test phylogenetic markers as an alternative to the use of rRNA genes, whose limited sequence divergence does not allow the accurate discrimination of diatoms at the species level. In this work, nine diatom species belonging to eight genera, isolated from epylithic environmental samples collected in central Italy, were chosen to implement a panel of diatoms covering the full range of ecological status of freshwaters. The procedure described in this work relies on the PCR amplification of specific regions in two conserved diatom genes, elongation factor 1-a (eEF1-a) and silicic acid transporter (*SIT*), as a first step to narrow down the complexity of the targets, followed by microarray hybridization experiments. Oligonucleotide probes with the potential to discriminate closely related species were designed taking into account the genetic polymorphisms found in target genes. These probes were tested, refined and validated on a small-scale prototype DNA chip. Overall, we obtained 17 highly specific probes targeting eEF1-a and *SIT*, along with 19 probes having lower discriminatory power recognizing at the same time two or three species. This basic array was validated in a laboratory setting and is ready for tests with crude environmental samples eventually to be scaled-up to include a larger panel of diatoms. Its possible use for the simultaneous detection of diatoms selected from the classes of water quality identified by the European Water Framework Directive is discussed.

## 1. Introduction

Diatoms are microscopic unicellular algae characterized by the unique ability to engineer a mixture of silica, proteins and carbohydrates to form complex silica shells with well-defined architectural elements. These photosynthetically active organisms contribute significantly to both terrestrial and marine primary productivity [1,2]. In addition to their enormous generation of organic carbon [3], diatoms play an ecologically relevant role in the global cycling of phosphorous, nitrogen and silica [4,5,6]. Due to their ability to colonize all habitats where a sufficient dose of light is available, and to respond to changes of environmental parameters such as temperature, pH, salinity, organic and inorganic nutrients, diatoms have been considered excellent ecological indicators. Analysis of freshwater diatom communities over the last 50 years has generated a comprehensive set of data that correlate the presence/absence of selected diatom species with the ecological status of rivers and lakes [7,8]. Several relevant studies have been conducted with the aim to correlate the quality of rivers and lakes through the use of indices based on diatoms [9,10,11,12,13].

The Water Framework Directive [14,15] provides a detailed road-map for the use of biological indicators, among which benthic diatoms, for the monitoring of water bodies. This Directive identifies five ecological status classes (high, good, moderate, poor and bad quality) by comparison with water environments subjected to minimal modifications by anthropogenic factors. In light of this approach, it is necessary to identify a panel of diagnostic diatoms to be used as water quality indicators and to develop robust methods for their identification.

Current methods for identification of freshwater diatoms rely on procedures based on light, epifluorescence or electron microscopic examination of their frustules, which consist of amorphous silica cell wall. However, to distinguish subtle morphological differences of frustules of different species can be very time consuming, and requires great taxonomic expertise. Moreover, many diatoms are relatively similar in size and shape leading to misidentification when morphologically closely-related species are compared. Therefore, there is a need for molecular methods that are rapid, simple and highly specific for the detection and identification of diatoms [16,17].

Microarrays are diagnostic tools consisting of ordered matrices of DNA sequences spotted on glass slides, which are used for hybridization experiments with fluorescently labelled target genes. This technology, which enables the parallel analysis of many genes in a single reaction, has been extensively used in both basic and applied aspects of the biological sciences. Microarrays have been applied to microbial ecology [18,19,20] in a series of studies involving harmful dinoflagellates of the genus *Alexandrium* [21,22], analysis of the prokaryotic diversity in soil extracts and organic wastes [23,24], and in the identification of pathogens [25,26]. The reference target gene used in the studies mentioned above was the small-subunit ribosomal RNA (SSU rRNA). While the use of this gene offers obvious advantages such as: (i) large amount of DNA sequence records available in the databases; (ii) the natural amplification of the transcribed gene; (iii) possibility to label directly total RNA extracted from cell cultures, there are nevertheless practical constraints. In fact, the limited sequence divergence of SSU rRNA in most of the cases allows the design of discriminatory oligonucleotide probes down to the genus level, under the best conditions, but does not allow for the possibility to distinguish organisms at the species level or strain level. In the case of diatoms, these limitations concern the overall assessment of phylogeny [27]. To overcome this restraint, researchers have explored the use of alternative genes as a source of species-specific genetic variability. In this context, the alternative genetic markers used were: *rpoB* [28], *gyrA* [29,30], *gyrB* [31,32], *iap* gene encoding protein p60 [33], genes for toxins [34,35], *groEL* [36], coding and non-coding regions of the chloroplast genome [37,38,39] and microsatellites [40].

We recently demonstrated that two marker genes, namely elongation factor 1-a (eEF1-a) and silicic acid transporter (*SIT*) can be successfully used for detection of a potential toxin-producing diatom species [41]. In this study, we have used the same strategy as an alternative to ribosomal RNA genes for the development of oligonucleotide probes capable of recognizing a panel of nine diatom species. The molecular probes based on the two marker genes were tested in microarray experiments with the aim to assemble a prototype suitable for the simultaneous and specific detection of diatom species representative of different water quality levels.

## 2. Materials and Methods

### 2.1. Environmental Sampling

**Figure 1:**
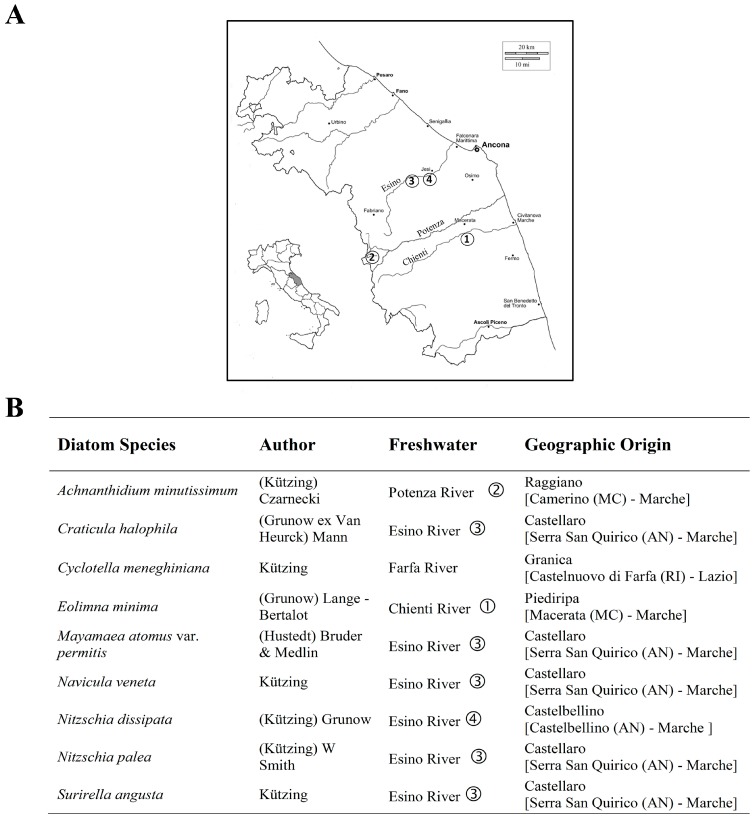
(**A**) Map of sampling sites in Regione Marche (Italy) and (**B**) list of diatoms species isolated and used in this work.

Environmental water samples containing diatoms were taken from four different locations, from the Chienti, Potenza and Esino Rivers in the Marche Region of Central Italy (Figure 1) over the period February 2011–February 2014. An additional freshwater sample, collected from the Farfa River, in the Lazio Region, was kindly provided by the Istituto Superiore di Sanità (ISS, National Institute of Health, Rome, Italy) during the same period. According to standard sampling methods [42], epilithic diatom cells were obtained by scraping with a toothbrush the surface of stones and rocks collected randomly in at least five different submerged sites of the rivers. Samples were stored in the dark at 4 °C until they were processed.

### 2.2. Diatoms Isolation and Culture Conditions

Nine diatom species were isolated directly from different freshwater samples. Individual diatoms were obtained by picking single cells from 30–50 μL of original sample monitored by an inverted microscope (Axiovert 25, Zeiss, Oberkochen, Germany), using a Gilson pipette equipped with a narrow tip. Once isolated, each diatom cell was inoculated in 20 mL of f/2 medium [43] and incubated under 12 h/12 h light-dark cycles of illumination at 18 °C constant temperature. Subsequently, the culture was transferred to 600 mL canted neck tissue culture flasks (Becton Dickinson Falcon, Le Pont De Claix, France). Nine pure diatom species were morphologically identified by light microscopy and maintained in the Laboratory of Genetics of the University of Camerino (Italy). The cultured species are listed in Figure 1. Eight out of nine diatom cultures that were used in this study were non-axenic species. In fact, the growth of bacteria of the genus *Pseudomonas* and *Staphylococcus* was detected by plating aliquots of diatom cultures on R2A agar medium (Oxoid, Thermo Fisher Scientific Brand, Waltham, MS, USA). The cell densities of the diatom cultures were monitored daily by use of a hemocytometer counting chamber (Bürker, Saaringia, Germany) and an inverted light microscope at either 10× or 20× magnification. Diatom cells in the exponential growth phase (*i.e.*, fourteen ‒ twenty days of incubation) were harvested by scraping the bottom of the culture flask followed by vacuum filtration (0.45 μm filter, Millipore, Millerica, MS, USA) and washing with 50 mL of sterile distilled water to eliminate the residual components of the f/2 medium. Filters were used for DNA extraction.

### 2.3. Morphological Identification

Living diatom cells were observed using the Zeiss Axiovert 25 light microscope at 20× magnification. Light microscope photographs were taken with a DSC-W730 Cyber-Shot camera (Sony, Tokyo, Japan). Pure diatom cultures were initially identified following Cox’s manual [44], a guide for the identification of common freshwater diatoms from living material. A more elaborate analysis was provided by colleagues at the Istituto Superiore di Sanità (ISS, National Institute of Health) in Rome. Organic substances were removed with hot hydrogen peroxide and HCl to yield a suspension of clean frustules, which were mounted as permanent slides in Naphrax^®^ (Brunel Microscopes, Chippenham, United Kingdom). The frustules were measured (length and width) and taxonomically identified to genus and species level, using reference texts reporting morphological criteria [45,46,47] and the software OMNIDIA [48].

### 2.4. Isolation of DNA and DNA Amplification

Chromosomal DNA was extracted from pure cultures of all nine diatom species as described elsewhere [17,41]. Specific DNA amplification of sequences of the two marker genes was achieved using the primer pair UniE-F (5’-ATCGAACAACACAAGAAGGT) and UniE-R (5’-ACCTTTCCA AGCATCTTCAA) targeting a 440 bp fragment of Elongation Factor gene (eEF1-a) and the primer pair UniS-F_1_ (5’-GACTTCATCAACAACTACTTCG) and UniS-R_1_ (5’-ACGTCCAATCATGAATCC AG) targeting a 470 bp fragment of the *SIT* gene. Forward primer UniS-F_2_ (5’-GACTWCATYAAC AACTACTTCG), containing two degenerate nucleotides proved more efficient compared to UniS-F_1_ for amplification of *SIT* from genomic DNA of *Eolimna minima* and *Navicula veneta*. Specific DNA amplification of the 190 bp fragment of SSU rRNA was obtained with primer 528F (5’-GCGGTAATT CCAGCTCCAA) and 650R (5’-AACACTCTAATTTTTTCACAG). The amount of chromosomal DNA used in DNA amplification reactions was in the range 2–100 ng. The experimental details of PCR conditions used have been described [41]. In most of the cases, to obtain the *SIT* fragment of interest (470 bp) it was necessary to purify from agarose gel the DNA amplification products. The DNA fragments obtained at the end of amplification and purification pathway were subjected to DNA sequencing using the same forward and reverse primers needed for DNA amplification.

### 2.5. Probe Design and Application of Microarray Experiments to Pure Diatom Cultures

The software tools used to design species-specific oligonucleotide probes are described elsewhere [41]. Common parameters selected for the layout of the oligonucleotide sequences were: melting temperature = 58–64 °C, length = 25–40 nt, minimum base pairs required for single primer self-dimerization = 4 bp, minimum base pair required for hairpin = 3 bp, GC content = 40%–60%. The OligoCalc tool was used for checking the properties of designed primers [49]. Microarray experiments were performed at Scienion AG (Berlin, Germany) following a protocol developed *ad hoc* for the analysis of microorganisms found in environmental samples. The procedure consists of a series of steps: printing, labelling, hybridization, washing, scanning and analyses of the results. The target used in these hybridization experiments consisted of 200 ng of the amplified DNA fragments (eEF1-a and *SIT* genes) obtained from pure diatom cultures. Following purification and determination of the DNA sequence, the amplicons were labelled using a Platinum *Bright*^TM^ 647 Infrared Nucleic Acid Labeling Kit (KREATECH Diagnostics, Amsterdam, The Netherlands) that binds a fluorophore molecule to the N^7^ position of guanines. Experimental conditions used in hybridization experiments and set-up of the array format are described elsewhere [41].

Fluorescence scanning of each slide was performed with a microarray scanner for the spots corresponding to samples and Cy3-marker, at 635 nm and at 532 nm, respectively. Images were analyzed by the software Array-Pro^®^ Analyzer. Subsequently, a grid of individual circles defining the name of each single dot and its position was aligned on the array and was used to correlate the fluorescence signal intensities with each dot of the array. Based on the estimation of microarray signals, which were averaged over eight individual spots, GPR files (GenePix^®^ Result) were generated and analyzed using GPR-Analyzer [50]. This software uses positive control probe intensity to normalize automatically the dataset. In fact, the signal derived from the control probes, complementary to a known amount of spiked DNA fragment corresponding to TATA box Binding Protein (TBP), provides a direct measure of hybridization efficiency. Hybridization signals were considered reliable if at least six spots in each block produced a fluorescent signal.

After three rounds of an iterative process of probe design, test, screening, removal of medium/low performance oligonucleotides and re-design, we finally obtained the MicroAqua-array-02 list consisting of 131 probes. The list of sequences of oligonucleotide probes is available in Supplementary Materials (Table S1, reporting the list of probe sequences for eEF1-a and *SIT* genes and Table S2 reporting the list of all probes spotted on the array).

## 3. Results

### 3.1. Isolation and Identification of Diatom Cells from Environmental Samples

Over the last 40 years, extensive data have been accumulated on the structure of the diatom population in rivers of the Central-East Apennine area of Italy [51,52]. These analyses, based on common criteria used by algologists to correlate physical and biological parameters with diatom assemblages [53,54], revealed a direct link between the presence and abundance of a selected panel of reliable diatom species and the water quality levels.

**Figure 2:**
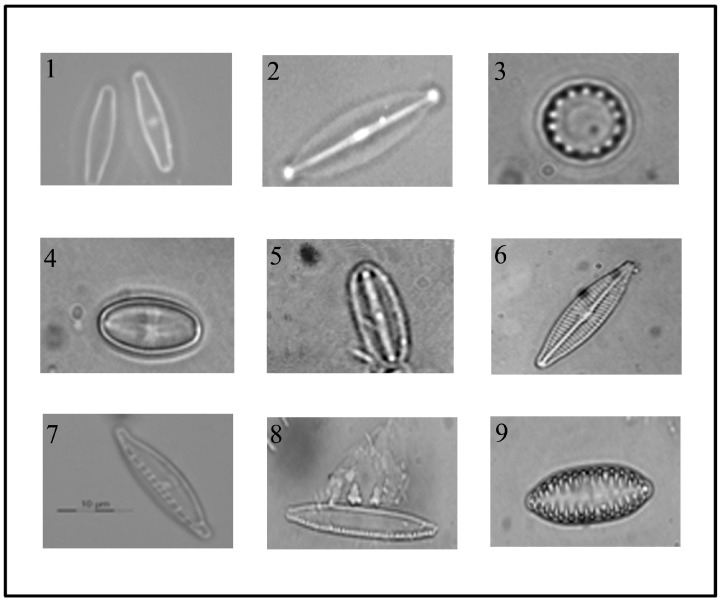
Photographs of the nine diatoms species used in this work: 1. Achnanthidium minutissimum; 2. Craticula halophila; 3. Cyclotella meneghiniana; 4. Eolimna minima; 5. Mayamaea atomus var. permitis; 6. Navicula veneta; 7. Nitzschia dissipata; 8. Nitzschia palea; 9. Surirella angusta. The pictures of diatom’s frustules were taken after removal of organic substances.

In this study, nine diatom species were selected to form a core panel that could serve as a frame of reference for identification of these bioindicators based on molecular methods (Figure 2). Epilithic water samples were collected in three rivers at sites selected by taking into account the different levels and impacts of anthropogenic activities on freshwater environments (Table 1).

The field samples were used to isolate single diatom cells required for the establishment of monoclonal cultures, which became part of the culture collection maintained at the laboratory of Genetics of the University of Camerino (Camerino, Italy). One centric diatom, *Cyclotella meneghiniana* Kützing, and eight pennate diatoms, namely *Achnanthidium minutissimum* (Kützing) Czarnecki, *Craticula halophila* (Grunow ex Van Heurck) Mann, *Eolimna minima* (Grunow) Lange-Bertalot, *Mayamaea atomus* var. *permitis* (Hustedt) Bruder & Medlin, *Navicula veneta* Kützing, *Nitzschia dissipata* (Kützing) Grunow, *Nitzschia palea* (Kützing) W. Smith and *Surirella angusta* Kützing were identified taxonomically by light microscopy analysis (Figure 2).

**Table 1:** Panel of nine diatom species and their correlation with water quality levels.

Diatom Species	Water Quality Levels According to EPI-D Index	Water Quality Levels According to ICMI Index	
		Index of Sensitivity to Pollutants (IPS_S)	Trophic Index (TI_TW)
1. *Achnanthidium minutissimum* (Kützing) Czarnecki	**I–II** **Excellent–Good**	**5** **Good–High**	**1,2** **Good–High**
2. *Craticula halophila* (Grunow ex Van Heurck) Mann	**IV–V** **Bad–Very Bad**	**2** **Bad–Poor**	**3,4** **Bad–Poor**
3. *Cyclotella meneghiniana* (Kützing)	**II–III** **Good - Mediocre**	**2** **Bad–Poor**	**2,8** **Poor–Sufficient**
4. *Eolimna minima* (Grunow) Lange-Bertalot	**IV** **Bad**	**2,2** **Poor–Sufficient**	**2,9** **Poor–Sufficient**
5. *Mayamaea atomus* var. *permitis* (Hustedt) Bruder & Medlin	**IV–V** **Bad–Very Bad**	**2,3** **Poor–Sufficient**	**3,1** **Bad–Poor**
6. *Navicula veneta* (Kützing)	**IV–V** **Bad–Very Bad**	**1** **Bad–Poor**	**3,5** **Bad–Poor**
7. *Nitzschia dissipata* (Kützing) Grunow	**II–III** **Good–Mediocre**	**4,5** **Good–High**	**2,4** **Sufficient–Good**
8. *Nitzschia palea* (Kützing) W Smith	**IV** **Bad**	**1** **Bad–Poor**	**3,3** **Bad–Poor**
9. *Surirella angusta* Kützing	**III** **Mediocre**	**4** **Sufficient–Good**	**3,7** **Bad–Poor**

Diatoms are representative of various water quality levels, which generally go from level V as very bad quality of water up to level I for an excellent water quality. To correlate each diatom species of the panel with its ecological distribution and its presence in a specific freshwater quality level, we selected two ecological indices, EPI-D index and ICMI index [52,55]. As indicated in Table 1, the panel of nine diatoms covers the full range of water quality levels; as a result, at least one diatom species for each level of water quality is available for either nucleic acid extraction or preparation of fresh cell cultures.

### 3.2. DNA Amplification and Sequencing of Conserved Diatom Genes

**Figure 3:**
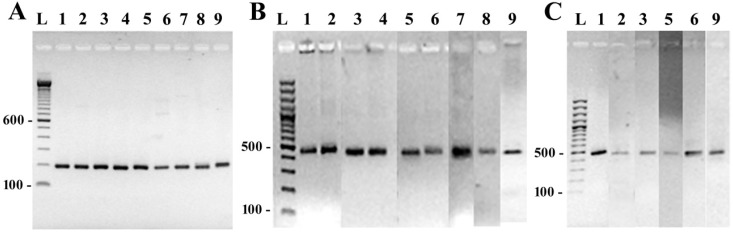
Electrophoretic separation of the amplification products obtained with primer pairs: (**A**) specific for SSU rRNA; (**B**) specific for a portion (440 bp) of the gene coding for the diatom elongation factor eEF1-a and (**C**) specific for a portion (470 bp) of the gene coding for the diatom silicic acid transporter gene (*SIT*). The panel of chromosomal DNA templates used corresponds to: 1. *Nitzschia dissipata*; 2. *Nitzschia palea*; 3. *Navicula veneta*; 4. *Surirella angusta*; 5. *Craticula halophila*; 6. *Eolimna minima*; 7. *Achnanthidium minutissimum*; 8. *Mayamaea atomus* var. *permitis* and 9. *Cyclotella meneghiniana*. Lane L contains the 100 bp DNA ladder Invitrogen (Panel A) and 100 bp DNA ladder Fermentas (Panel B and C).

Although the traditional methods of diatom identification based on microscopy analysis are well established, we sought to devise a molecular approach for the scrutiny of diatom population structure. As a first step, we used an optimized DNA extraction method to isolate from all nine cultures genomic DNA, which was tested for use in PCR-based DNA amplification reactions. To test reliability of the system, a preliminary amplification was carried out using primers targeting the small-subunit ribosomal RNA. The presence of a reproducible and strong amplicon confirmed the quality of the extracted material (Figure 3, panel A).

Next, we applied to the whole panel of diatoms a strategy for the identification, screening and application of oligonucleotide probes that could be used as species-specific markers. To reach this goal, we followed an experimental protocol successfully devised for the development of oligonucleotide probes directed at the identification of the potential toxin producer diatom species *Amphora coffeaeformis* (Agardh) Kützing [41]. Universal primers targeting the two conserved genes eEF1-a and the diatom-specific gene *SIT* were used to prepare a set of fragments for all nine species of the panel by DNA amplification.

The DNA fragments obtained at the end of amplification and purification pathway are shown in Figure 3, panels B and C. As a result of this preliminary phase, we obtained a collection of original DNA sequence data pertaining to a region of *SIT* and eEF1-a for five and nine diatom species, respectively (Table S3, Supplementary Materials).

### 3.3. Species-Specific Oligonucleotide Probe Design

The DNA sequences described in Section 3.2 were aligned, compared with the sequences of two marine diatoms (*Thalassiosira pseudonana* and *Phaeodactylum tricornutum*), available in the databases, and used to design oligonucleotides that may potentially act, *in silico*, as species-specific probes. In light of the fact that this study was carried out within the framework of a European collaborative project aimed at the development of microarrays for the detection of pathogenic microorganisms present in freshwaters (e.g., bacteria, cyanobacteria, protozoa and viruses) and diagnostic diatom species, the strategy used for probe design was based on the principle that all oligonucleotides should have very similar melting temperatures (Tm = 58‒64 °C). This is an absolute requirement since the microarray format should be amenable for the simultaneous detection of all the above mentioned organisms present in freshwater samples.

**Figure 4:**
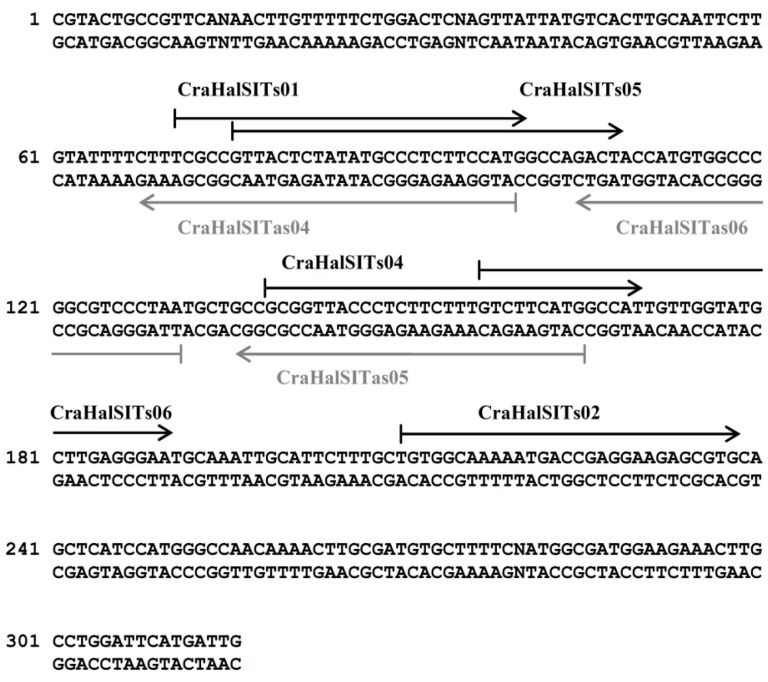
Details of oligonucleotide probes designed on the amplicon corresponding to a portion of *SIT* gene of *Craticula halophila*. Black and grey arrows indicate sense probes and antisense probes, respectively.

As a general rule, at least six probes complementary to both DNA strands were designed for each of the diatom marker genes eEF1-a and *SIT*. A representation of the outcome of this procedure is shown in Figure 4, where five probes (CraHalSITs01, CraHalSITs02, CraHalSITs04, CraHalSITs05 and CraHalSITs06) designed on the sense strand and three probes (CraHalSITas04, CraHalSITas05 and CraHalSITas06) designed on the antisense strand are displayed on the sequence of *Craticula halophila SIT*.

### 3.4. Hybridization Experiments on Microarrays

The procedure described in Section 3.3 yielded for both marker genes a provisional compilation of oligonucleotide sequences that was termed MicroAqua-array-00. To test the efficiency of the preliminary set of molecular probes, oligonucleotides were spotted on glass slides microarrays, which were used, in combination with the fluorescently labelled DNA amplicons of each gene of the panel of diatoms, for hybridization experiments.

As expected, the first round of microchip experiments yielded essentially three responses, namely: (i) highly specific probes, (ii) probes reacting with both their specific DNA target and with non-specific amplicons and (iii) non-specific probes. It was therefore necessary to return to the design step, modify some of the common parameters and prepare a revised version of the list of oligonucleotide sequences, which consisted in the highly specific probes and the *quasi*-specific probes, together with the *de-novo* designed probes replacing the non-specific ones. The new version of the MicroAqua-array was experimentally tested on microarray hybridization experiments.

**Figure 5:**
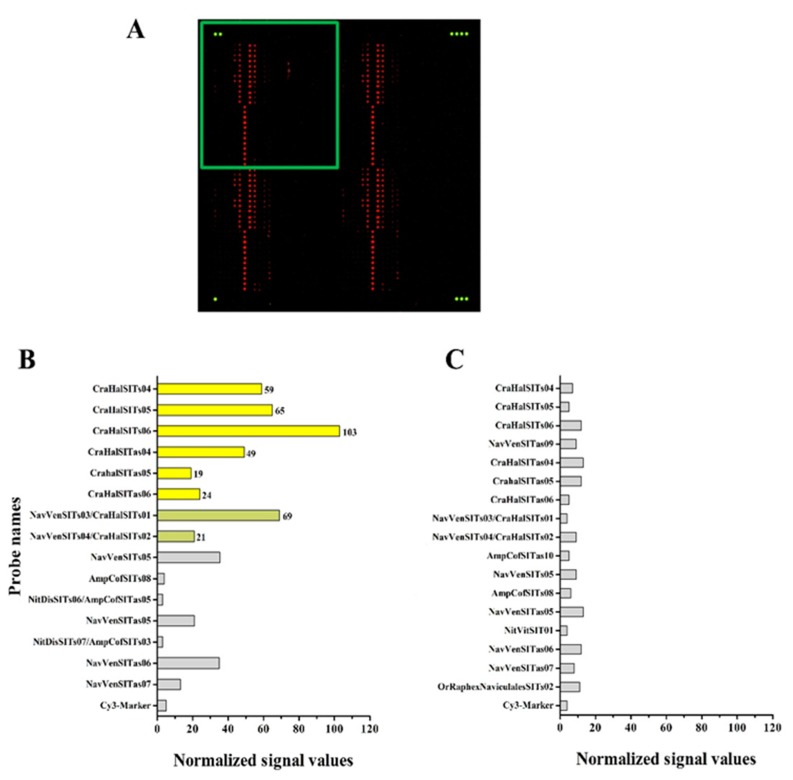
Results of microarray hybridization experiments targeting *SIT* gene. (**A**) Image of an hybridized slide after scanning: the green dots indicate the Cy3-Marker spotted at the four corners of the slide, the green square indicates one of the four sectors of each block of the array. In the two graphs are reported the values of normalized signals obtained after hybridization with a fluorescent fragment of *SIT* gene from *Craticula halophila* (**B**) and *Nitzschia palea* (**C**). On the Y-axis are reported the probes producing a fluorescent signal after hybridization: the color code of the bars follows the scheme reported in Table S1 and in the text. Grey bars indicate false-positive signals.

**Figure 6:**
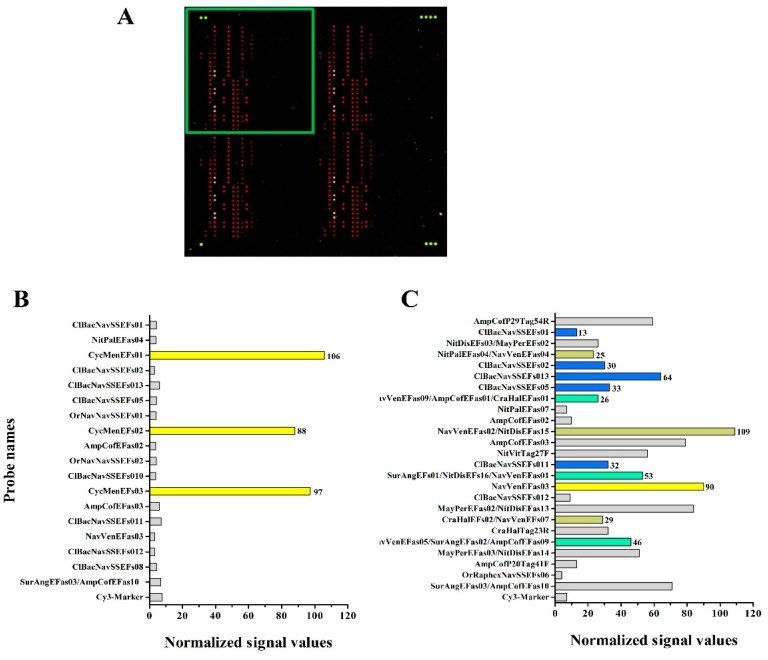
Results of microarray hybridization experiments targeting eEF1-a gene. (**A**) Image of an hybridized slide after scanning: the green dots indicate the Cy3-Marker spotted at the four corners of the slide, the green square indicates one of the four sectors of each block of the array. In the two graphs are reported the values of normalized signals obtained after hybridization with a fluorescent fragment of eEF1-a gene from *Cyclotella meneghiniana* (**B**) and *Navicula veneta* (**C**). On the Y-axis are reported the probes producing a fluorescent signal after hybridization: the color code of the bars follows the scheme reported in Table S1 and in the text. Grey bars indicate false-positive signals.

After three rounds of an iterative process of probe design, test, screening, removal of medium/low performance oligonucleotides and design of new probes, we finally obtained the MicroAqua-array-02 consisting of 131 probes (Table S2, Supplementary Material). A representative image of the results of microarray experiments after conversion of fluorescent signals into color-coded spots, is shown in Figure 5 and Figure 6. In the hybridization experiment shown in Figure 5 (Panel B), the DNA chip was tested with a labelled DNA fragment of *SIT* derived from *Craticula halophila*. In this case, four probes (CraHalSITs04, CraHalSITs05, CraHalSITs06 and CraHalSITas04) produced strong and specific signals (yellow bars), two probes (CraHalSITas05 and CraHalSITas06) displayed lower fluorescence intensities, but still very specific (yellow bars) and two probes (NavVenSITs03/CraHalSITs01 and NavVenSITs04/CraHalSITs02), identified by green bars produced a signal also with a target derived from *Navicula veneta*.

In a similar way, the hybridization experiment carried out with a labelled DNA fragment of eEF1-a derived from *Cyclotella meneghiniana* produced very strong and specific signals for three probes (CycMenEFs01, CycMenEFs02 and CycMenEFs03) identified by yellow bars in Figure 6, Panel B.

Some of the hybridization experiments carried out with purified target DNA revealed also cross-hybridization signals, as indicated in panel C of Figure 5 (the target used was *SIT* of *Nitzschia palea*) and in panel C of Figure 6 (the target used was eEF1-a of *Navicula veneta*). These non-specific signals are either weak (grey bars in Figure 5, panel C) or correspond to a limited number of probes (light green, dark green and blue bars in Figure 6, panel C) recognized by non-target species.

We assume that when the DNA chip produces a specific, strong response with at least 60% of the whole set of probes designed for a diatom species and most of the background signals (grey bars) are well below the threshold value, it is reasonable to state that the output of the microarray demonstrates the presence of the given diatom in the sample. In addition, using this microarray format it is possible to carry out tandem determination analyses for this panel of diatom species (based on presence/absence of signals for two unrelated genes) which, compared to single-gene analyses, can achieve considerably higher levels of accuracy.

Overall, a total of 19 probes targeting eEF1-a and 17 probes targeting *SIT* of the nine diatom species have been developed, tested and validated in this work. In Table 2 are summarized the oligonucleotide probes available for each species of the panel of diatoms, for each of the two marker genes used in this study.

**Table 2:** Summary of the discriminatory power displayed by oligonucleotide probes targeting two marker genes in microarray hybridization experiments.

Diatom Species	Target (eEF1-a)	Target (*SIT*)	Total Probes
*Achnanthidium minutissimum*	3 *		**3**
*Craticula halophila*	2 *	6 + 2 *	**10**
*Cyclotella meneghiniana*	3		**3**
*Eolimna minima*		3	**3**
*Mayamaea permitis*	1 *		**1**
*Navicula veneta*	1 + 1 *		**2**
*Nitzschia dissipata*		6 *	**6**
*Nitzschia palea*	2 *		**2**
*Surirella angusta*	4 + 2 *		**6**

* Denotes probes reacting with both their specific DNA target and with non-specific amplicons.

## 4. Discussion and Conclusions

The aim of this study was to develop molecular tools for the identification of freshwater diatoms representative of the five different water quality levels indicated by the European Union’s Water Framework Directive (WFD). We focused our attention on a panel of nine diatoms species routinely found in rivers of the Central-East Apennine area, for which extensive population data were available [51,52].

This study was carried out within the collaborative “Universal Microarrays for the evaluation of fresh-water Quality based on detection of pathogens and their toxins” (MicroAQUA) project sponsored by the EU 7th Framework Programme. The aim of MicroAQUA was to develop an efficient, sensitive and robust test for detecting and quantifying toxic algae, pathogenic bacteria and protozoa, viruses and toxins in freshwaters. In addition to recognizing pathogenic microorganisms and viruses possibly contaminating the waters, the DNA chip will also facilitate the assessment of their ecological status by detecting the presence/absence of diatom species previously identified as water quality indicators. Therefore, as a preliminary step, it was necessary to set-up a culture collection of pure diatom species isolated from freshwater samples and to generate a diatom DNA bank consisting of chromosomal DNA extracted from diatom cultures. Nine freshwater diatom species were selected as a core-panel based on their reliability as bioindicators (Table 1).

The main advantage of microarray technology is the capacity to test a large number of probes in a single hybridization experiment with target RNA or DNA samples. Depending on the marker genes employed and the strategy used for the design of the probes, microarrays can provide resolution at various taxonomic levels down to species or even strain level. Here we describe the construction of a diagnostic oligonucleotide microarray for the rapid and simultaneous detection of the core-panel of diatom species.

We had previously demonstrated that microarray technology is amenable for identification of freshwater diatoms [56]. However, the use of ribosomal RNA genes does not provide sufficient resolution to distinguish all diagnostic diatoms at the species level [27]. Thus, we examined alternative genes as a source of raw material required to attain the necessary specificity for probe design. As described in the accompanying article [41], at the end of a screening process two conserved genes coding for elongation factor (eEF1-a) and the diatom-specific silicic acid transporter (*SIT*) were chosen to develop oligonucleotide probes suitable for application on microarrays. Notably, it was found that production of eEF1-a DNA fragment was relatively easy in most of the cases, while the amplification of *SIT* frequently resulted in the simultaneous generation of nonspecific DNA fragments. This might be due, at least in part, to the presence of multiple copies of *SIT* gene that may subtract primers from binding the specific target. It should also be taken into account that the DNA sequence recognized by the “universal” primers targeting the two marker genes may vary among different species and the presence of one or a few mismatches can have profound effects on DNA yield.

The rationale for using this integrated approach was both the exploration of new genetic markers among highly conserved genes and the possibility to perform identification analyses with improved accuracy and resolution [57]. The results obtained in this study indicate that the two target genes were found to possess sufficient polymorphic information to discriminate related microorganisms. This discrimination power is further reinforced by the combined use of probes designed on both marker genes.

Overall, the procedure involved a series of hybridization experiments where the probes were tested, analyzed, removed in case they produced cross-hybridization results, designed in a different position and re-analyzed in a cyclic screening system required to confirm the specificity of duplex formation of the designed probes with their corresponding and specific target amplicons.

In agreement with previous observations, discrimination between specific and a-specific signals is a major challenge in DNA microarray-based species detection. In fact, the ability to predict by bioinformatic tools the hybridization behavior of the oligonucleotide probes is still approximate and requires improvement. This is especially true for short probes, typically in the range of 15–40 nucleotides. In our experience, many of the probes validated *in silico* in the preliminary analyses were recognized by non-target species in hybridization tests. Cross-hybridization reactions were observed in a large number of cases, therefore it was necessary to design, test and redesign several probes for each diatom species. Thus, starting from a large number of oligonucleotides it was possible to obtain, after many trials, a restricted but specific panel of probes. These species-specific oligonucleotide probes can be used for detecting target species representative of different water quality levels (Table 1) and can be regarded as the starting tool on which additional probes may be added in the future for the throughput detection of many other diatoms.

Microarrays were originally devised to analyze simultaneously the amount of mRNA transcripts from many genes. While gene expression profiling is focused on the comparison of two well defined biological systems (e.g., model systems or cell lines), the use of genotyping arrays for recognition of organisms in environmental samples poses new and unique challenges. In fact, it is well known that in ecological microarray studies, the kind of DNA isolation method applied and the PCR amplification strategy can significantly affect the accuracy of the entire procedure of microorganism identification. , Further studies are required to determine if the diatom chip containing the probe-set developed in this work (MicroAqua-array-02) can detect the presence of diagnostic diatoms when tested with complex environmental samples. Preliminary assays based on the use of spiked diatom cell mixtures and field-testing are currently ongoing in our laboratory. As the outcomes of these studies become available, they will provide a solid background to test SaDA, a software developed in a parallel study for rapid and automated management and analysis of ecological high-throughput data [58].

As a result of advances in genomics technologies, new methods for microbial identification have been recently proposed. One entails the use of long oligonucleotide probes (50-mer or 60-mer) targeting *in silico* identified marker genes and it has been applied to the identification of human microbiome and bacteria of clinical relevance [59,60]. In the absence of complete genome sequences of freshwater diatoms, this type of high-resolution microchip test cannot be applied now, but may represent a promising perspective. In another approach, the variations in the nuclear-encoded internal transcribed sequence (ITS), a potentially high-resolution phylogenetic marker, were explored for recognition of diatoms at the species level. This approach, based on DNA-barcoding, relies on the differences of the DNA sequence in a small portion (300‒400 bp) of the ribosomal RNA operon [61]. However, to date no attempt has been made to convert this information into a systematic design of oligonucleotide probes suitable for DNA chip technology tests.

Although many studies have corroborated the idea that diatom-based indexes can be directly correlated with chemical data and the presence of pollutants, more recent analyses have demonstrated that, in several cases, there is a weak relationship between the index values and the ecological status of rivers [11,62,63]. Furthermore, many major issues like (i) the choice of the metric for water quality assessment, (ii) the reliability of some diagnostic diatoms for different ecoregional water types, (iii) the time required by the diatom assemblages to adapt to water quality changes and iv) the exact boundaries between the five ecological status classes identified by the European Union’s WFD, are still left unsolved.

This study is intended to provide an advanced molecular tool for the simultaneous analysis of complex diatom mixtures. The objective was to construct a device that can provide a highly sensitive response on the presence/absence of diatoms adapted to living in environments characterized by different water quality levels. This should help, at least in part, to bridge the gap between the evidence of the widespread abundance of these essential elements at the basis of the aquatic food chain, and a more defined assignment of their role as freshwater bioindicators.

## Supplementary Files

Supplementary File 1
