# Supplementary material for: Molecular Tools for the Selective Detection of Nine Diatom Species Biomarkers of Various Water Quality Levels
Source: Int J Environ Res Public Health. 2015 May 22;12(5):5485–504. doi: 10.3390/ijerph120505485 (PMC4454981; doi:10.3390/ijerph120505485)
Supplement: Supplementary File 1 [file ijerph-12-05485-s001.pdf]

Supplementary Information

Molecular Tools for the Selective Detection of Nine Diatom Species Biomarkers of Various Water Quality Levels

---

Table S1. Sequence of the oligonucleotide probes developed in this work. The probe's classification is based on the two marker genes (eEF1-a and *SIT*), on the diatom species and also on the position within the target DNA sequence (sense strand or anti-sense strand).

**Elongation Factor Gene (eEF1-a)**			
**Diatom Species**	**Probe Name**		**Probe Sequence 5' → 3'**
Achnanthidium minutissimum (Kützing) Czarnecki	**Antisense strand**	**AchMinEFas01**	ATACCGACAGAGTTTCCGGGTCCGGCAG
		**AchMinEFas03**	ACAGGACAGTATCCAACCTTGAGAACTCCGGG
		**AchMinEFas04**	CGCGGTACGGGAAAAGATAACAGGGCAGTAT
		**AchMinEFas05**	CAACCTTCTGTCCTCCAGTCTTCTTCGATTGC
		**AchMinEFas02**	GCAACCTTGGCGGTACGGCAGAAAATAACAG
Craticula halophila (Grunow ex Van Heurck) Mann	**Sense strand**	**CraHalEFs02**	TATTGCCAAGGACGAGAAGGTACAGCC
	**Antisense strand**	**CraHalEFas05**	GGCTGGACCTTCTCTTCCTTGGCAATA
		**CraHalEFas01**	CTTCCACAAAATCTTGGTCATCTTGCA
Cyclotella meneghiniana Kützing	**Sense strand**	**CycMenEFs01**	CTTGTGGAAGATGGGCAAGAAGACTGGAGG
		**CycMenEFs02**	ACTCCTGTTGTCTTCTGCCGTACTGCCAA
		**CycMenEFs03**	AAGATGAGAAGGTTGAGCCAGGTGACA
Mayamaea atomus var. *permitis* (Hustedt) Bruder & Medlin	**Sense strand**	**MayPerEFs01**	TTATTTTCTGCCGTACCGCCAAGGTTGC
		**MayPerEFs02**	CTGTCGGTATGTCCATCAGGGGTATTGCCAAG
		**MayPerEFs03**	AGAAGACCGGAGGTGCCAAGTTGGAAAATCCC
	**Antisense strand**	**MayPerEFas02**	ATGTCTCCAGGCTGTACCTTCTCGTCCTT
		**MayPerEFas03**	TTATCGACCTTGGCACCTCCCGTCTTCTT
		**MayPerEFas04**	ATACCGACAGAGTTTCCGGGTCCGGCAG
		**MayPerEFas05**	ACAGGACAGTATCCAACCTTGAGAACTCCGGG
		**MayPerEFas06**	GCAACCTTGGCGGTACGGCAGAAAATAACAG
Navicula veneta Kützing	**Sense strand**	**NavVenEFs07**	TATTGCCAAGGACGAGAAGGTACAGCC
	**Antisense strand**	**NavVenEFas07**	GTCAACCTTGGCACCTCCAGTCTTCTT
		**NavVenEFas08**	GACCTTCTCGTCCTTTCCAATACCCTTGA
		**NavVenEFas03**	GGCTTGAGGACACCGGGATGCTCTTGGAC
		**NavVenEFas02**	AAGGCAGTGAACGACTTGACGGGCTTGAG
		**NavVenEFas04**	GGGCAGTATCCGGGCTTGAGGACACCA
		**NavVenEFas01**	GGCTGTACCTTCTCGTCCTTGGCAATA
		**NavVenEFas05**	GCTGTACCTTCTCGTCCTTGGCAATAC
		**NavVenEFas09**	CTTCCACAAAATCTTGGTCATCTTGCA
Nitzschia dissipata (Kützing) Grunow	**Sense strand**	**NitDisEFs02**	TTATTTTCTGCCGTACCGCCAAGGTTGC
		**NitDisEFs03**	CTGTCGGTATGTCCATCAGGGGTATTGCCAAG
		**NitDisEFs04**	AGAAGACCGGAGGTGCCAAGTTGGAAAATCCC
	**Antisense strand**	**NitDisEFas04**	GGCTGGACCTTCTCTTCCTTGGCAATA
		**NitDisEFas13**	ATGTCTCCAGGCTGTACCTTCTCGTCCTT
		**NitDisEFas14**	TTATCGACCTTGGCACCTCCCGTCTTCTT
		**NitDisEFas15**	AAGGCAGTGAACGACTTGACGGGCTTGAG
		**NitDisEFas06**	ATACCGACAGAGTTTCCGGGTCCGGCAG
		**NitDisEFas16**	GGCTGTACCTTCTCGTCCTTGGCAATA
		**NitDisEFas07**	ACAGGACAGTATCCAACCTTGAGAACTCCGGG
		**NitDisEFas08**	GCAACCTTGGCGGTACGGCAGAAAATAACAG

Table S1. *Cont.*

Elongation Factor Gene (eEF1-a)			
**Diatom Species**	**Probe Name**		**Probe Sequence 5' → 3'**
Nitzschia palea (Kützing) W Smith	**Sense strand**	**NitPalEFs02**	AAGGACGAAAAGGTCAACCCCGGAGACATCATC
		**NitPalEFs03**	CTCTGTTGGTATGTCCGTCAAGGGTATTGGAAAGG
		**NitPalEFs04**	CCTCAAGCCTGGATACTGTCCCATCATTTTCTC
		**NitPalEFs05**	TTTCACCGCTTTGGTCGCTGTTCAGGAG
		**NitPalEFs06**	CATCAAGTCTTTCACCGCTTTGGTCGCTG
		**NitPalEFs07**	GTCGCTGTTCAGGAGCACCCCGGTGT
	**Antisense strand**	**NitPalEFas06**	ACTGGGAAAGTTCTGGGGGGTTGTCGACCTT
		**NitPalEFas07**	GAAAATGATGGGACAGTATCCAGGCTTGAGGACAC
		**NitPalEFas08**	GTCTCCGGGGTTGACCTTTTCGTCCTTTCCAATA
		**NitPalEFas01**	GACCTTCTCGTCCTTTCCAATACCCTTGA
		**NitPalEFas02**	GTCAACCTTGGCACCTCCAGTCTTCTT
		**NitPalEFas04**	GGGCAGTATCCGGGCTTGAGGACACCA
		**NitPalEFas05**	CAACCTTGGCACCTCCAGTCTTCTTCGA
		**NitPalEFas09**	CGCGGTACGGGAAAAGATAACAGGGCAGTAT
		**NitPalEFas09**	CAACCTTCTGTCCTCCAGTCTTCTTCGATTGC
Surirella angusta Kützing	**Sense strand**	**SurAngEFs01**	ACAGGACAGTATCCAACCTTGAGAACTCCGGG
		**SurAngEFs04**	GAAGGTTCTTGACTCCGCCGGACCAGGAA
		**SurAngEFs05**	TACTGCCCTGTTATCTTTTCCCGTACCGCG
		**SurAngEFs06**	GGAAGCAATCGAAGAAGACTGGAGGACAGAAG
	**Antisense strand**	**SurAngEFas01**	GGCTGTACCTTCTCGTCCTTGGCAATA
		**SurAngEFas02**	GCTGTACCTTCTCGTCCTTGGCAATAC
		**SurAngEFas03**	AATCTTGGTCATCTTGCAGGCAACCTTGGC
		**SurAngEFas06**	GCAACCTTGGCGGTACGGCAGAAAATAACAG
		**SurAngEFas07**	AACAGGGCAGTATCCCGGCTTGAGGACAC
		**SurAngEFas04**	CGCGGTACGGGAAAAGATAACAGGGCAGTAT
		**SurAngEFas05**	CAACCTTCTGTCCTCCAGTCTTCTTCGATTGC
**Bacillariophyceae**	**OrNavNavSSEFs01**		AGCGACCAAAGCGGTGAAAGACTTGATGG
	**OrNavNavSSEFs02**		CCAGGGTTGACCTTCTCATCCTTGGCAATACC
	**OrNavNavSSEFas01**		TTTCACCGCTTTGGTCGCTGTTCAGGAG
	**OrNavNavSSEFas02**		CATCAAGTCTTTCACCGCTTTGGTCGCTG
	**OrNavNavSSEFas03**		GTCGCTGTTCAGGAGCACCCCGGTGT
	**ClBacNavSSEFs01**		GCAAGATGACCAAGATTCTCTGGAAGCAGTCC
	**ClBacNavSSEFs02**		GTTATCTTCTCTCGTACCGCCAAGGTTGCCT
	**ClBacNavSSEFs03**		GCCCGGTTACTGTCCTGTTATCTTCTCTCGTAC
	**ClBacNavSSEFs04**		GACTGCTTCCAGAGAATCTTGGTCATCTTG
	**ClBacNavSSEFs05**		AGGCAACCTTGGCGGTACGAGAGAAGATAA
	**ClBacNavSSEFs06**		TACGAGAGAAGATAACAGGACAGTAACCGGGC
	**ClBacNavSSEFs08**		CAAGAAGACCGGTGGTGCCAAGGTTGACAA
	**ClBacNavSSEFs09**		CCCTGGTGACATCATCTACCTTGAAAAGGAAGG
	**ClBacNavSSEFs010**		TCAACCTTGGCACCACCGGTCTTCTTGGAC
	**ClBacNavSSEFs011**		GATAACAGGACAGTAACCGGGCTTGAGGACACC
	**ClBacNavSSEFs012**		CAACCTTGGCGGTACGAGAGAAGATAACAGGA
	**ClBacNavSSEFs013**		ATCTTGGTCATCTTGCAGGCAACCTTGGC

Table S1. *Cont.*

Silicic Acid Transporter Gene (*SIT*)			
**Diatom Species**	**Probe Name**		**Probe Sequence 5′ → 3′**
Craticula halophila (Grunow ex Van Heurck) Mann	**Sense strand**	**CraHalSITs09**	CATTCTTCTCCGTTACTGCCTGTTGCTC
		**CraHalSITs04**	GCGGTTACCCTCTTCTTTGTCTTCATGGCCAT
		**CraHalSITs05**	GTTACTCTATATGCCCTCTTCCATGGCCAGACT
		**CraHalSITs06**	GTCTTCATGGCCATTGTTGGTATGCTTGAGGGA
		**CraHalSITs01**	TCGCCGTTACTCTATATGCCCTCTTCCATG
		**CraHalSITs02**	TGTGGCAAAAATGACCGAGGAAGAGCGTG
	**Antisense strand**	**CraHalSITas08**	CCAACGGCGTCTCTGTATTCCTCTTCTT
		**CraHalSITas04**	ATGGAAGAGGGCATATAGAGTAACGGCGAAAG
		**CraHalSITas05**	CATGAAGACAAAGAAGAGGGTAACCGCGGC
		**CraHalSITas06**	TTAGGGACGCCGGGCCACATGGTAGTC
Eolimna minima (Grunow) Lange-Bertalot	**Sense strand**	**EolMinSITs02**	GATTCAGATCGCCTTCTTCGCCGTCACC
	**Antisense strand**	**EolMinSITas01**	GCGAAGAAGGCGATCTGAATCGCCTCGAGCAT
		**EolMinSITas02**	GGTGACGGCGAAGAAGGCGATCTGAATC
Navicula veneta Kützing	**Sense strand**	**NavVenSITs05**	GCATTGCTGCTATCTTGTACAGATGTTCTTCG
		**NavVenSITs01**	CCAACGGCGTCTCTGTATTCCTCTTCTT
		**NavVenSITs02**	CATTCTTCTCCGTTACTGCCTGTTGCTC
		**NavVenSITs03**	TCGCCGTTACTCTATATGCCCTCTTCCATG
		**NavVenSITs04**	TGTGGCAAAAATGACCGAGGAAGAGCGTG
	**Antisense strand**	**NavVenSITas05**	TGGCCATGGAAGAGGGCATATAGAGTAACGG
		**NavVenSITas06**	GGCGAAGAACATCTGTACAAGATAGCAGCAAT
		**NavVenSITas07**	ATGAAGACAAAGAAGAGGGTAACCGCGGCA
		**NavVenSITas09**	CACATGGTGGTCTGTCCATTGAACAGGG
		**NavVenSITas10**	CCAAAGAAGAAGATGTTTTGCATAGTGTT
Nitzschia dissipata (Kützing) Grunow	**Sense strand**	**NitDisSITs06/AmpCofSITas05**	CACCATGAGCACTCTCTTCAACGAACAAACCAC
		**NitDisSITs07/AmpCofSITs03**	AATCTCTTCTTCTGGGGACGATGCCTCTTCTCT
		**NitDisSITs08/AmpCofSITas09**	GCTGTTGCCAAGATCCGCGAGTCGGAA
	**Antisense strand**	**NitDisSITas02/AmpCofSITas02**	GCAAAAGCACACGACGGCGAAAGAGAAG
		**NitDisSITas03/AmpCofSITas03**	AGAAGAAGAGATTGGCCACGGCACTGC
		**NitDisSITas04/AmpCofSITas04**	AAGCACAATTCCCTTGGGGATTCCCTCCCA
Nitzschia palea (Kützing) W Smith	**Antisense strand**	**NitPalSITas11**	GAGCAACAGGCAGTAACGGAGAAGAATG
**Bacillariophyceae**	**OrRaphSITs01**		TTGCCGACATAGACTTCATCACGCTGGA
	**OrRaphexNaviculalesSITs02**		GGAACACCCTCCCACATGGTGGTCTGTCCA

Probes classification

Probe producing either weak or no signal
Species-specific probe
Probe recognizing two species
Probe recognizing three species
Probe recognizing four species
Probe recognizing Order level and Genus level diatoms
Probe recognizing Class level diatoms (Bacillariophyceae)

Table S2. List of oligonucleotides spotted on the MicroAqua-array-02. Multiple names indicate probes whose sequence is shared by more than one diatom species.

MicroAqua-array-02 Probe Name
Cy3-Marker
Buffer
Empty
AmpCofTag7F
AmpCofTag25
NitPalEFas02/NavVenEFas07
SurAngEFas03/AmpCofEFas10
OrRaphexNaviculalesSITs02
AmpCofSITas08
ClBacNavSSEFs09
NavVenSITas07
NitDisSITs08/AmpCofSITas09
SurAngEFas07
Lambda
AmpCofP20Tag41F
NitVitTag7R
NavVenEFas05/SurAngEFas02/AmpCofEFas09
MayPerEFas03/NitDisEFas14
EolMinSITas02
AmpCofSITas07
ClBacNavSSEFs08
NavVenSITas06
NitDisSITs07/AmpCofSITs03
SurAngEFas05/AchMinEFas05/NitPalEFas10
POSITIVE_25_dT_12.5-µM
NitDisTag46R
AmpCofTagP29
NitDisEFas04/CraHalEFas05
NavVenEFas05/SurAngEFas02/AmpCofEFas09
NitVitSIT01
AmpCofSITas06
ClBacNavSSEFs03
NavVenSITas05
NitDisSITs06/AmpCofSITas05
SurAngEFas04/AchMinEFas04/NitPalEFasS09
POSITIVE_25_dT_25-µM
CraHalTag23R
AmpCofP20Tag221R
CraHalEFs02/NavVenEFs07
MayPerEFas02/NitDisEFas13
EolMinSITs02
AmpCofSITs08
ClBacNavSSEFs012
NavVenSITs05
NitDisSITas04/AmpCofSITas04
SurAngEFs06
POSITIVE_25_dT_50 µM
AmpCofTag46F
NavVenTag55
NavVenEFas03
SurAngEFs01/NitDisEFs16/NavVenEFas01
AmpCofTag10
AmpCofSITs07
ClBacNavSSEFs011
NavVenSITs04/CraHalSITs02
NitDisSITas03/AmpCofSITas03
SurAngEFs05
DunGS05_25_dT_dT_12.5 µM
NitDisTag23F
NitVitTag27F
AmpCofEFas03
CycMenEFs03
EolMinSITas01
AmpCofSITs06
ClBacNavSSEFs010
NavVenSITs03/CraHalSITs01
NitDisSITas02/AmpCofSITas02
SurAngEFs04
DunGS05_25_dT_dT_25 µM
NitPalTag36F
NitVitTag7
NavVenEFas02/NitDisEFas15
NitPalEFas01/NavVenEFas08
NitPalSITas11
ClBacNavSSEFs06
CraHalSITas06
OrNavNavSSEFs02
NitDisEFas08/SurAngEFas06/AchMinEFas02/MayPerEFas06
NitPalEFas08
ClBacNavSSEFs03
DunGS05_25_dT_dT_50-µM
AmpCofTag5R
AmpCofEFas02
CycMenEFs02
NavVenSITas10
NavVenSITs01/CraHalSITas08
CrahalSITas05
OrNavNavSSEFs01
NitDisEFas07/SurAngEFs01/AchMinEFas03/MayPerEFas05
NitPalEFas07
DunGS02_25_dT_dT_12.5 µM
NitDisTag67R
NitPalTag23
NavVenEFas09/AmpCofEFas01/CraHalEFas01
NitPalEFas05/AmpCofEFas11
OrRaphSITs01
ClBacNavSSEFs05
CraHalSITas04
ClBacNavSSEFs013
NitDisEFas06/AchMinEFas01/MayPerEFas04
NitPalEFas06
ClBacNavSSEFs02
DunGS02_25_dT_dT_25 µM
AmpCofP20F
AmpCofTag
CycMenEFs01
NavVenSITas09
CraHalSITs06
OrNavNavSSEFas03/NitPalEFs07
NitDisEFs04/MayPerEFs03
NitPalEFs04
DunGS02_25_dT_dT_50 µM
NitDisTag67F
NitDisTag7R
SurAngEFs01/NitDisEFs16/NavVenEFas01
NitPalEFas04/NavVenEFas04
AmpCofTag09
ClBacNavSSEFs04
CraHalSITs05
OrNavNavSSEFas02/NitPalEFs06
NitDisEFs03/MayPerEFs02
NitPalEFs03
ClBacNavSSEFs01
euk 1209
AmpCofTag29R
AmpCofP29Tag54R
AmpCof29Tag
CraHalSITs04
OrNavNavSSEFas01/NitPalEFs05
NitDisEFs02/MayPerEFs01
NitPalEFs02
NavVenSITs02/CraHalSITs09
euk 328

Table S3. Collection of DNA sequences of diatom marker genes obtained in this work.

Diatom Species	Silicic Acid Transporter Gene (*SIT*) [Sequence 5' → 3']
Craticula halophila	1 CGTACTGCCGTTCANAACTTGTTTTTCTGGACTCNAGTTATTATGTCACTTGCAATTCTT<br>GCATGACGGCAAGTNTTGAACAAAAAGACCTGAGNTCAATAATACAGTGAACGTTAAGAA<br><br>61 GTATTTTCTTTCGCCGTTACTCTATATGCCCTCTTCCATGGCCAGACTACCATGTGGCCC<br>CATAAAAGAAAGCGGCAATGAGATATACGGGAGAAGGTACCGGTCTGATGGTACACCGGG<br><br>121 GGCGTCCCTAATGCTGCCGCGGTTACCCTCTTCTTTGTCTTCATGGCCATTGTTGGTATG<br>CCGCAGGGATTACGACGGCGCCAATGGGAGAAGAAACAGAAGTACCGGTAACAACCATAC<br><br>181 CTTGAGGGAATGCAAATTGCATTCTTTGCTGTGGCAAAAATGACCGAGGAAGAGCGTGCA<br>GAACTCCCTTACGTTTAACGTAAGAAACGACACCGTTTTTACTGGCTCCTTCTCGCACGT<br><br>241 GCTCATCCATGGGCCAACAAAACTTGCGATGTGCTTTTCNATGGCGATGGAAGAAACTTG<br>CGAGTAGGTACCCGGTTGTTTTGAACGCTACACGAAAAGNTACCGCTACCTTCTTTGAAC<br><br>301 CCTGGATTCATGATTG<br>GGACCTAAGTACTAAC
Eolimna minima	1 GTTTCCAGAGAAGATCCCCGAACCGGCTTTGNGAAGGTCTTCTTCTGGGCTCGGTGCGTC<br>CAAAGGTCTCTTCTAGGGGCTTGGCCGAAACNCTTCCAGAAGAAGACCCGAGCCACGCAG<br><br>61 CTCTCGATCGTTCTCTCGGGCGTTGCGTTCGCGGTCATCATGGCGGGCCTCGTCGAGGGA<br>GAGAGCTAGCAAGAGAGCCCGCAACGCAAGCGCCAGTAGTACCGCCCGGAGCAGCTCCCT<br><br>121 AAGACCACCGTGTGGGAGGGCGTCCCTCATTGGGCGGCGATTATCGTCTTCTTTGTATTC<br>TTCTGGTGGCACACCCTCCCGCAGGGAGTAACCCGCCGCTAATAGCAGAAGAAACATAAG<br><br>181 GCGGGCCTCGTTGGGATGCTCGAGGCGATTCAGATCGCCTTCTTCGCCGTCACCGAAGTC<br>CGCCCGGAGCAACCCTACGAGCTCCGCTAAGTCTAGCGGAAGAAGCGGCAGTGGCTTCAG<br><br>241 ACCACGGCCGAACGCGGGAACGCCAAGTTCGCGATGATGACGTGCGAG<br>TGGTGCCGGCTTGCGCCCTTGCGGTTCAAGCGCTACTACTGCACGCTC
Navicula veneta	1 GTGACTTCATTAACAACTACTTCGGCTATTTCACCTTGCAAGTGTCAAGAGTGATTGAAT<br>CACTGAAGTAATTGTTGATGAAGCCGATAAAGTGGAACGTTCACAGTTCTCACTAACTTA<br><br>61 GGTCCGGTCTGTTGCATTGCTGCTATCTTGTACAGATGTTCTTCGCCAAGATCTCTGGCC<br>CCAGGCCAGACAACGTAACGACGATAGAACATGTCTACAAGAAGCGGTTCTAGAGACCGG<br><br>121 AGGGACTCGAAACAAAGGAAGAACCCCGTACTGCCGTTCAGAACTTGTTTTTCTGGACTC<br>TCCCTGAGCTTTGTTTCCTTCTTGGGGCATGACGGCAAGTCTTGAACAAAAAGACCTGAG<br><br>181 GAGTTATTATGTCACTTGCAATTCTTGTATTTTCTTTCGCCGTTACTCTATATGCCCTCT<br>CTCAATAATACAGTGAACGTTAAGAACATAAAAGAAAGCGGCAATGAGATATACGGGAGA<br><br>241 TCCATGGCCAGACTACCATGTGGCCCGGCGTCCCTAATGCTGCCGCGGTTACCCTCTTCT<br>AGGTACCGGTCTGATGGTACACCGGGCCGCAGGGATTACGACGGCGCCAATGGGAGAAGA<br><br>301 TTGTCTTCATGGCCATTGTTGGTATGCTTGAGGGAATGCAAATTGCATTCTTTGCTGTGG<br>AACAGAAGTACCGGTAACAACCATACGAACTCCCTTACGTTTAACGTAAGAAACGACACC<br><br>361 CAAAAATGACCGAGGAAGAGCGTGCAGCTCATCCATGGGCCAAGAAAACTGCGA<br>GTTTTTACTGGCTCCTTCTCGCACGTCGAGTAGGTACCCGGTTCTTTTGACGCT

Table S3. *Cont.*

Diatom Species	Silicic Acid Transporter Gene (*SIT*) [Sequence 5' → 3']
Nitzschia dissipata	1 GAATTCTCCGGACTGTTGCATGCTTCCTACCTCCTTGAAATGATGGTTTGCAGCATGGCT<br>CTTAAGAGGCCTGACAACGTACGAAGGATGGAGGAACTTTACTACCAAACGTCGTACCGA<br><br>61 GGACAACCCAAGGTTAGCAACGAACCCCCTCGCAGTGCCGTGGCCAATCTCTTCTTCTGG<br>CCTGTTGGGTTCCAATCGTTGCTTGGGGGAGCGTCACGGCACCGGTTAGAGAAGAAGACC<br><br>121 GGACGATGCCTCTTCTCTTTCGCCGTCGTGTGCTTTTGCGCGGCCGTCACCATGAGCACT<br>CCTGCTACGGAGAAGAGAAAGCGGCAGCACACGAAAACGCGCCGGCAGTGGTACTCGTGA<br><br>181 CTCTTCAACGAACAAACCACCGTCTGGGAGGGAATCCCCAAGGGAATTGTGCTTGTCCTT<br>GAGAAGTTGCTTGTTTGGTGGCAGACCCTCCCTTAGGGGTTCCCTTAACACGAACAGGAA<br><br>241 TTCGTGGTGTTGTTCTGCGTCGTTGGTTTGTTGGAGGCCATGCAAATCGCCTTTTTCGCT<br>AAGCACCACAACAAGACGCAGCAACCAAACAACCTCCGGTACGTTTAGCGGAAAAAGCGA<br><br>301 GTTGCCAAGATCCGCGAGTCGGAACG<br>CAACGGTTCTAGGCGCTCAGAATTGC
Nitzschia palea	1 GCAACTTTGGTGGCCCTGTTCAATGGACAGACCACCATGTGGGAGGGTGTTCCCAACGGT<br>CGTTGAAACCACCGGGACAAGTTACCTGTCTGGTGGTACACCCTCCCACAAGGGTTGCCA<br><br>61 GTCGCCGTCCTTGTTTTCTTCCTTCTTATGGCGGTCGTAGGAATGTTGGAAGGAATGCAA<br>CAGCGGCAGGAACAAAAGAAGGAAGAATACCGCCAGCATCCTTACAACCTTCCTTACGTT<br><br>121 ATCGCCTTTTTNTCTGTATCCNGAATCCCCGAGGATGAACGTGGNGACTCCTNGANTGCC<br>TAGCGGAAAAANAGACATAGGNCTTAGGGGCTCCTACTTGCACCNCTGAGGANCTNACGG<br><br>181 AAGAAAGTANGCAA<br>TTCTTTCATNCGTT

Table S3. *Cont.*

Diatom Species	Elongation Factor Gene (eEF1-a) [Sequence 5' → 3']
Achnanthidium minutissimum	1 TCCGCCGGACCCGGAAACTCGGTTGGTATGTCCATTAGTGGTATTGCCAAGGAAGAGAAG<br>AGGCGGCCTGGGCCTTTGAGCCAACCATACAGGTAATCACCATAACGGTTCCTTCTCTTC<br><br>61 GTCCAGCCCGGAGATATCATTTACGTCGAGAAGGAAGGTGAACTCAAGCCCGTCAAGGCT<br>CAGGTCGGGCCTCTATAGTAAATGCAGCTCTTCCTTCCACTTGAGTTCGGGCAGTTCCGA<br><br>121 TTCACCGCCATGGTCGCCGTCCAGGAACACCCCGGAGTGCTCAAGGTCGGATACTGTCCT<br>AAGTGGCGGTACCAGCGGCAGGTCCTTGTGGGGCCTCACGAGTTCCAGCCTATGACAGGA<br><br>181 GTTATTTTCTGCCGTACCGCCAAGGTTGCCTGCAAGATGACCAAGATCTTGTGGAAGCAG<br>CAATAAAAGACGGCATGGCGGTTCCAACGGACGTTCTACTGGTTCTAGAACACCTTCGTC<br><br>241 TCCAAGAAGACCGGAGGTGCCAAGTTGGAGAACCCCCCGGAACTGTCACAGTACGAAAAC<br>AGGTTCTTCTGGCCTCCACGGTTCAACCTCTTGGGGGGCCTTGACAGTGTCATGCTTTTG<br><br>301 GCTGAAGTCGAATTCGAACCCACCGCCCCCTTGTTCGTCGAGCCCTTTGAGGTCTGTGCA<br>CGACTTCAGCTTAAGCTTGGGTGGCGGGGGAACAAGCAGCTCGGGAAACTCCAGACACGT
Craticula halophila	1 CACAAGAAGGTCCTTGACTCGGCCGGACCAGGAAACTCGGTCGGCATGTCCATCAAGGGT<br>GTGTTCTTCCAGGAACTGAGCCGGCCTGGTCCTTTGAGCCAGCCGTACAGGTAGTTCCCA<br><br>61 ATTGCCAAGGACGAGAAGGTACAGCCTGGAGACATTATCTACCTTGAAAAGGAAGGTCAC<br>TAACGGTTCCTGCTCTTCCATGTCGGACCTCTGTAATAGATGGAACTTTTCCTTCCAGTG<br><br>121 CTCAAGCCCGTCAAGTCGTTCACTGCCTTTGTCGCCGTCCAAGAGCATCCCGGTGTCCTC<br>GAGTTCGGGCAGTTCAGCAAGTGACGGAAACAGCGGCAGGTTCTCGTAGGGCCACAGGAG<br><br>181 AAGCCTGGTTACTGCCCTGTCATTTTCTCTCGTACCGCCAAGGTTGCCTGCAAGATGACC<br>TTCGGACCAATGACGGGACAGTAAAAGAGAGCATGGCGGTTCCAACGGACGTTCTACTGG<br><br>241 AAGATTTTGTGGAAGCAGTCCAAGAAGACGGGAGGNGCCAAGGTCGATAACCCTCCCGAA<br>TTCTAAAACACCTTCGTCAGGTTCTTCTGCCCTCCNCGGTTCCAGCTATTGGGAGGGCTT<br><br>301 CTGTCTCAGTTTGAATCCGCCGAGGTCGAATTCGAACCCACTGCCCCCTTGTTCTTGGAA<br>GACAGAGTCAAACTTAGGCGGCTCCAGCTTAAGCTTGGGTGACGGGGGAACAAGAACCTT<br><br>361 CCCTTTGAGACATGTGCCGC<br>GGGAAACTCTGTACACGGCG
Cyclotella meneghiniana	1 GTCCAGAAAGAGGGAGAACTCAAGCCCATTAAGTCTTTCACTGCCATGGTTGCCGTTCAA<br>CAGGTCTTTCTCCCTCTTGAGTTCGGGTAATTCAGAAAGTGACGGTACCAACGGCAAGTT<br><br>61 GAACATCCTGGTGTCCTGAAGCCTGGTTACACTCCTGTTGTCTTCTGCCGTACTGCCAAA<br>CTTGTAGGACCACAGGACTTCGGACCAATGTGAGGACAACAGAAGACGGCATGACGGTTT<br><br>121 GTTGCTTGCAAGATGACCAAGATCTTGTGGAAGATGGCCAAGAAGACTGGAGGGCAGAAG<br>CAACGAACGTTCTACTGGTTCTAGAACACCTTCTACCGGTTCTTCTGACCTCCCGTCTTC<br><br>181 GTAGAGAATCCCCCGGAACTTTCCCAATATGAGAATGCTGAGGTTGAGTTCGTACCAAGT<br>CATCTCTTAGGGGGCCTTGAAAGGGTTATACTCTTACGACTCCAACTCAAGCATGGTTCA<br><br>241 CAGCCACTCTTTGTTGAGCCCTTTGAAACATGTGCGGCTCTTGGACGTATTGCTGTTATG<br>GTCGGTGAGAAACAACTCGGGAAACTTTGTACACGCCGAGAACCTGCATAACGACAATAC<br><br>301 GATTCCAAC<br>CTAAGGTTG

Table S3. *Cont.*

Diatom Species	Elongation Factor Gene (eEF1-a) [Sequence 5′ → 3′]
Eolimna minima	1 CACAAGAAGGTCCTTGACTCGGCCGGACCAGGAAACTCGGTCGGCATGTCCATCAAGGGT<br>GTGTTCTTCCAGGAACTGAGCCGGCCTGGTCCTTTGAGCCAGCCGTACAGGTAGTTCCCA<br><br>61 ATTGCCAAGGACGAGAAGGTACAGCCTGGAGACATTATCTACCTTGAAAAGGAAGGTCAC<br>TAACGGTTCCTGCTCTTCCATGTCGGACCTCTGTAATAGATGGAACTTTTCCTTCCAGTG<br><br>121 CTCAAGCCCGTCAAGTCGTTCACTGCCTTTGTCGCCGTCCAAGAGCATCCCGGTGTCCTC<br>GAGTTCGGGCAGTTCAGCAAGTGACGGAAACAGCGGCAGGTTCTCGTAGGGCCACAGGAG<br><br>181 AAGCCTGGTTACTGCCCTGTCATTTTCTCTCGTACCGCCAAGGTGGCCTGCAAGATGACC<br>TTCGGACCAATGACGGGACAGTAAAAGAGAGCATGGCGGTTCCACCGGACGTTCTACTGG<br><br>241 AAGATTTTGTGGAAGCAGTCCAAGAAGACGGGAGGTGCCAAGGTCGATAACCCTCCCGAA<br>TTCTAAAACACCTTCGTCAGGTTCTTCTGCCCTCCACGGTTCCAGCTATTGGGAGGGCTT<br><br>301 CTGTCTCAGTTTGAATCCGCCGAGGTCGAATTCGAACCCACTGCCC<br>GACAGAGTCAAACTTAGGCGGCTCCAGCTTAAGCTTGGGTGACGGG
Mayamaea permitis	1 CAAGAAGGTCCTTGAATCCGCCGGACCCGGTAACTCCGTTGGTCTTTCCATCAAGGGTAT<br>GTTCTTCCAGGAACTTAGGCGGCCTGGGCCATTGAGGCAACCAGAAAGGTAGTTCCCATA<br><br>61 TGCCAAGGATGAGAAGGTCAACCCTGGTGACATCATCTACCTTGAAAAGGAAGGTGAACT<br>ACGGTTCCTACTCTTCCAGTTGGGACCACTGTAGTAGATGGAACTTTTCCTTCCACTTGA<br><br>121 CAAGCCCATCAAGTCTTTCACCGCTTTGGTCGCTGTTCAGGAGCACCCCGGTGTCCTCAA<br>GTTCGGGTAGTTCAGAAAGTGGCGAAACCAGCGACAAGTCCTCGTGGGGCCACAGGAGTT<br><br>181 GCCCGGTTACTGTCCAGTCATCTTCTCTCGTACCGCCAAGGTTGCCTGCAAGATGACCAA<br>CGGGCCAATGACAGGNCAGTAGAAGAGAGCATGGCGGTTCCAACGGACGTTCTACTGGTT<br><br>241 GATCCTCTGGAAGCAGTCCAAGAAGACCGGTGGTGCCAAGGTTGACAACCCTCCGGAACT<br>CTAGGAGACCTTCGTCAGGTTCTTCTGGCCACCACGGTTCCAACTGTTGGGAGGCCTTGA<br><br>301 CTCGCAGTT<br>GAGCGTCAA
Navicula veneta	1 GACTCGGCCGGACCAGGAAACTCGGTCGGCATGTCCATCAAGGGTATTGCCAAGGACGAG<br>CTGAGCCGGCCTGGTCCTTTGAGCCAGCCGTACAGGTAGTTCCCATAACGGTTCCTGCTC<br><br>61 AAGGTACAGCCTGGAGACATTATCTACCTTGAAAAGGAAGGTCACCTCAAGCCCGTCAAG<br>TTCCATGTCGGACCTCTGTAATAGATGGAACTTTTCCTTCCAGTGGAGTTCGGGCAGTTC<br><br>121 TCGTTCACTGCCTTTGTCGCCGTCCAAGAGCATCCCGGTGTCCTCAAGCCTGGTTACTGC<br>AGCAAGTGACGGAAACAGCGGCAGGTTCTCGTAGGGCCACAGGAGTTCGGACCAATGACG<br><br>181 CCCGTCATTTTCTCTCGTACCGCCAAGGTGGCCTGCAAGATGACCAAGATTTTGTGGAAG<br>GGGCAGTAAAAGAGAGCATGGCGGTTCCACCGGACGTTCTACTGGTTCTAAAACACCTTC<br><br>241 CAGTCCAAGAAGACGGGAGGTGCCAAGGTCGATAACCCTCCCGAACTGTCTCAGTTTGAA<br>GTCAGGTTCTTCTGCCCTCCACGGTTCCAGCTATTGGGAGGGCTTGACAGAGTCAAACTT<br><br>301 TCCGCCGAGGTCGAATTCGAACCCACTGCCNNCCTTGTTCTTGGAACCT<br>AGGCGGCTCCAGCTTAAGCTTGGGTGACGGNNGGAACAAGAACCTTGGA

Table S3. *Cont.*

Diatom Species	Elongation Factor Gene (eEF1-a) [Sequence 5' → 3']
Nitzschia dissipata	1 AGAAGGTCCTTGACTCTGCCGGACCCGGAAACTCTGTCGGTATGTCCATCAGGGGTATTG<br>TCTTCCAGGAACTGAGACGGCCTGGGCCTTTGAGACAGCCATACAGGTAGTCCCCATAAC<br><br>61 CCAAGGAAGAGAAGGTTCAGCCCGGAGATATCATCTACGTCGAAAAGGAGGGTGAACTCA<br>GGTTCCTTCTCTTCCAAGTCGGGCCTCTATAGTAGATGCAGCTTTTCCTCCCACTTGAGT<br><br>121 AGCCCGTCAAGAGTTTCACTGCCATGGTCGCCGTCCAGGAACACCCCGGAGTTCTCAAGG<br>TCGGGCAGTTCTCAAAGTGACGGTACCAGCGGCAGGTCCTTGTGGGGCCTCAAGAGTTCC<br><br>181 TTGGATACTGTCCTGTTATTTTCTGCCGTACCGCCAAGGTTGCGTGCAAAATGACCAAGA<br>AACCTATGACAGGACAATAAAAGACGGCATGGCGGTTCCAACGCACGTTTTACTGGTTCT<br><br>241 TCTTGTGGAAGCAGTCCAAGAAGACCGGAGGTGCCAAGTTGGAAAATCCCCCGGAACTGT<br>AGAACACCTTCGTCAGGTTCTTCTGGCCTCCACGGTTCAACCTTTTAGGGGGCCTTGACA<br><br>301 CACAGTACGAAAACGCCGAAGTCGAATTCGAACCCACTGCCCCCTTGTTCGTCGA<br>GTGTCATGCTTTTGCGGCTTCAGCTTAAGCTTGGGTGACGGGGGAACAAGCAGCT
Nitzschia palea	1 TTATCGAACAACACAAGAAGGTTCTTGACTCCGCCGGACCAGGAAACTCTGTTGGTATGT<br>AATAGCTTGTTGTGTTCTTCCAAGAACTGAGGCGGCCTGGTCCTTTGAGACAACCATACA<br><br>61 CCGTCAAGGGTATTGGAAAGGACGAAAAGGTCAACCCCGGAGACATCATCTTCCTGGAGA<br>GGCAGTTCCCATAACCTTTCCTGCTTTTCCAGTTGGGGCCTCTGTAGTAGAAGGACCTCT<br><br>121 AGGAGGGTGCATTGAAGCCCATCAAGTCCTTCACTGCCCTTGTTGCCGTCCAAGAGCACC<br>TCCTCCCACGTAACTTCGGGTAGTTCAGGAAGTGACGGGAACAACGGCAGGTTCTCGTGG<br><br>181 CTGGTGTCCTCAAGCCTGGATACTGTCCCATCATTTTCTCACGTACCGCCAAAGTTGCGT<br>GACCACAGGAGTTCGGACCTATGACAGGGTAGTAAAAGAGTGCATGGCGGTTTCAACGCA<br><br>241 GCAAAATGACCAAGATTCTTTGGAAGCAATCGAAGAAGACGGGAGGTGCCAAGGTCGACA<br>CGTTTTACTGGTTCTAAGAAACCTTCGTTAGCTTCTTCTGCCCTCCACGGTTCCAGCTGT<br><br>301 ACCCCCCAGAACTTTCCCAGTTCGAATCCGCCGAAGTTGAGTTCGAACCCACTGCCCCGC<br>TGGGGGGTCTTGAAAGGGTCAAGCTTAGGCGGCTTCAACTCAAGCTTGGGTGACGGGGCG<br><br>361 TTTTCGTCGAGCC<br>AAAAGCAGCTCGG
Surirella angusta	1 GAACAACACAAGAAGGTCCTTGACTCGGCCGGACCAGGAAACTCGGTCGGCATGTCCATC<br>CTTGTTGTGTTCTTCCAGGAACTGAGCCGGCCTGGTCCTTTGAGCCAGCCGTACAGGTAG<br><br>61 AAGGGTATTGCCAAGGACGAGAAGGTACAGCCTGGAGACATTATCTACCTTGAAAAGGAA<br>TTCCCATAACGGTTCCTGCTCTTCCATGTCGGACCTCTGTAATAGATGGAACTTTTCCTT<br><br>121 GGTCACCTCAAGCCCGTCAAGTCGTTCACTGCCTTTGTCGCCGTCCAAGAGCATCCCGGT<br>CCAGTGGAGTTCGGGCAGTTCAGCAAGTGACGGAAACAGCGGCAGGTTCTCGTAGGGCCA<br><br>181 GTCCTCAAGCCTGGTTACTGCCCTGTCATTTTCTCTCGTACCGCCAAGGTTGCCTGCAAG<br>CAGGAGTTCGGACCAATGACGGGACAGTAAAAGAGAGCATGGCGGTTCCAACGGACGTTC<br><br>241 ATGACCAAGATTTTGTGGAAGCAGTCCAAGAAGACGGGAGGTGCCAAGGTCGATAACCCT<br>TACTGGTTCTAAAACACCTTCGTCAGGTTCTTCTGCCCTCCACGGTTCCAGCTATTGGGA<br><br>301 CCCGAACTGTCTCAGTTTGAATCCGCCGAGGTCGAATTCGAACCCACTGCCCCCTTGTTC<br>GGGCTTGACAGAGTCAAACTTAGGCGCCTCCAGCTTAAGCTTGGGTGACGGGGGAACAAG<br><br>361 TTGGAACCCTTTGAGACATGTGCCGC<br>AACCTTGGGAAACTCTGTACACGGCG

© 2015 by the authors; licensee MDPI, Basel, Switzerland. This article is an open access article distributed under the terms and conditions of the Creative Commons Attribution license (http://creativecommons.org/licenses/by/4.0/).
